# Discerning the Role of Blood Brain Barrier Dysfunction in Alzheimer’s Disease

**DOI:** 10.14336/AD.2022.0130-1

**Published:** 2022-10-01

**Authors:** Qing-Qing Tao, Rong-Rong Lin, Yi-He Chen, Zhi-Ying Wu

**Affiliations:** Department of Neurology and Research Center of Neurology in Second Affiliated Hospital, and Key Laboratory of Medical Neurobiology of Zhejiang Province, Zhejiang University School of Medicine, Hangzhou, China.

**Keywords:** Alzheimer’s disease;, blood-brain barrier, amyloid-β, tau, neuroinflammation

## Abstract

Alzheimer’s disease (AD) is the most common form of neurodegenerative disease. The predominant characteristics of AD are the accumulation of amyloid-β (Aβ) and hyperphosphorylated tau in the brain. Blood brain barrier (BBB) dysfunction as one of the causative factors of cognitive impairment is increasingly recognized in the last decades. However, the role of BBB dysfunction in AD pathogenesis is still not fully understood. It remains elusive whether BBB dysfunction is a consequence or causative fact of Aβ pathology, tau pathology, neuroinflammation, or other conditions. In this review, we summarized the major findings of BBB dysfunction in AD and the reciprocal relationships between BBB dysfunction, Aβ pathology, tau pathology, and neuroinflammation. In addition, the implications of BBB dysfunction in AD for delivering therapeutic drugs were presented. Finally, we discussed how to better determine the underlying mechanisms between BBB dysfunction and AD, as well as how to explore new therapies for BBB regulation to treat AD in the future.

## 1. Introduction

Alzheimer’s disease (AD) is a complex disorder that is clinically characterized by the progressive decline in cognition, and pathologically characterized by the accumulation of amyloid-β (Aβ) and phosphorylated tau (P-tau) in the brain [1]. In recent years, a series of studies have demonstrated that AD is linked to blood brain barrier (BBB) dysfunction [2]. BBB dysfunction has been identified in the early stage of AD [3]. The BBB is a continuous membrane formed by a tightly sealed monolayer of endothelial cells. The main function of the BBB is maintenance of the brain health micro-environment by keeping neurotoxic components, pathogens, and circulating blood out of the brain [4]. The first clues regarding BBB dysfunction came from studies performed in AD genetic animal models with Aβ or tau pathology [5]. Therefore, at that time, it was believed that BBB dysfunction was associated with Aβ or tau pathology. However, BBB breakdown and vascular dysregulation were also determined in preclinical and early-stage AD patients before cognitive decline or positive Aβ and tau pathology [6]. These results suggested that the BBB breakdown that appeared in the early stage of AD could not be fully explained by the consequence of Aβ and/or tau pathology (the forms of plaques, tangles and oligomers). In recent years, emerging evidence has supported the contributions of neuroinflammation to AD pathogenesis [7]. The associations between BBB breakdown and neuroinflammation have been explored in several studies [8, 9]. An injured BBB was associated with neuroinflammation such as microglial activation and elevated inflammatory cytokines release [10, 11]. However, the exact role of BBB dysfunction in AD pathogenesis is still unknown. It remains elusive whether BBB dysfunction is a consequence or a cause of Aβ pathology, tau pathology, neuroinflammation, or other conditions.

In this review, we summarize the major findings of BBB dysfunction in AD and the pathogenic mechanisms by which BBB dysfunction results in AD onset and neurodegenerative processes, particularly the reciprocal relationships between BBB dysfunction, Aβ pathology, tau pathology, and neuroinflammation. Furthermore, the implications of BBB dysfunction for delivering AD therapeutic drugs are presented. Finally, we discuss future directions to better determine the underlying mechanisms between BBB dysfunction and AD and explore new therapies for BBB regulation to treat AD.

## 2. Molecular structure and functional characteristics of the BBB

**Figure 1.**
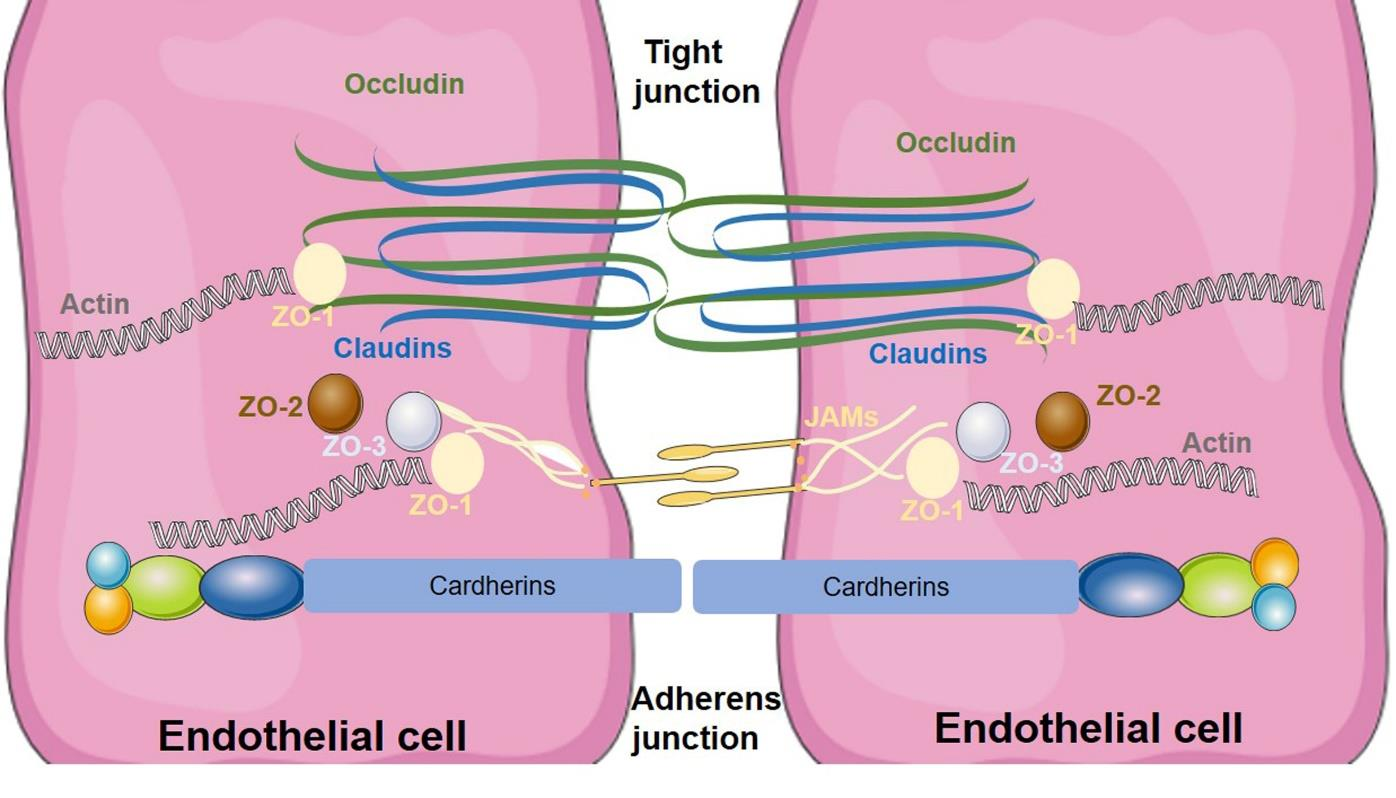
**Molecular characteristics of tight junction (TJ) protein complexes at the blood-brain barrier (BBB)**. The TJ is formed by several transmembrane proteins (claudins, occludin, and junction adhesion molecule) on adjacent endothelial cells. The C terminal of these transmembrane proteins is bound to cytoskeletal actin by ZO-1. The alteration of these proteins at the TJ may cause abnormal BBB permeability.

The BBB is a selective semipermeable border. Several cellular elements such as endothelial cells, astrocyte end-feet, and pericytes involve in the formation of BBB [12]. Endothelial tight junctions (TJs) between endothelial cells form the physical barrier [13]. Several essential membrane proteins, including claudin, occludin, and junction adhesion molecules, as well as cytoplasmic accessory proteins such as Zonula occludens protein1 (ZO-1), ZO-2, and ZO-3, participate in the formation of TJs (Fig. 1). Astrocyte end-feet are also integral to the formation of the BBB, which locate the abluminal aspect of endothelial cells. Astrocytes secrete nutritional factors that reciprocally interact with endothelial cells and help sustain the BBB [14]. Pericytes are cells located at the basement membrane of blood microvessels and wrap around endothelial cells, astrocytes, and neurons. The signal transduction pathways in pericytes have been reported to be involved in BBB permeability, angiogenesis, and neuroinflammation [15].

The BBB serves a critical role in the maintenance of central nervous system (CNS) homeostasis. It prevents the movement of pathogens, neurotoxins, immune factors, and large molecules from the peripheral blood into the cerebrospinal fluid (CSF) [16]. In addition, the BBB is not only a physical barrier but also serves as a selective transport boundary. Metabolic products such as glucose, amino acids, and nucleosides, can be actively transported across the barrier by specific transporters [17]. Another important structure related to the BBB and neurovascular function is the neurovascular unit (NVU). The NVU consists of endothelial cells, a basal lamina, pericytes, and different types of neural cells (neurons, astrocytes, and an extracellular matrix) [18]. All these cellular and extracellular components form a complex entity regulating cerebral blood flow (CBF) and BBB function.

## 3. Evidence of BBB dysfunction in AD

There are several aspects to BBB dysfunction: (1) alteration of BBB permeability, which causes circulating substances to enter the brain; (2) BBB transporter dysfunction, which results in the accumulation of neurotoxic substances in the brain and disturbance of energy and nutrient supplies; and (3) abnormality of secreted proteins from endothelial cells, astrocytes and other cellular elements of the BBB that can induce neuronal injury [19]. Under physiological conditions, the BBB has limited permeability that controls the transfer of substances from the peripheral circulation to the brain. Limited BBB permeability shields the brain from rapid changes in metabolic conditions and exposure to molecules that are harmful to neurons and other cells in the brain. BBB permeability can be affected by vascular endothelial cells, neurons, astrocytes, pericytes, and the extracellular matrix [20]. In addition, the BBB contains various membrane transporters facilitate the influx or efflux of amino acids, glucose, and nucleosides [21]. It is reported that BBB function is compromised during aging and AD. The literature on BBB dysfunction in AD has expanded over the last decade [22]. The evaluation of BBB dysfunction has been described using various methods, including neuroimaging studies, comparison of CSF/serum ratios of substances of peripheral origin (such as albumin), and neuropathological studies documenting evidence of blood proteins in the CNS [23, 24].

**Table 1:** BBB disruption on neuroimaging in human subjects.

Subjects	Strategies	Main findings	References
**Cognitively normal older adults**	DP-ASL-MRI	Low water exchange rate across the BBB is associated with low CSF Aβ42 concentration.	[37]
**MCI**	DCE-MRI	An age-dependent BBB breakdown in the hippocampus of MCI.	[31]
**MCI**	DCE-MRI	A smaller vascular volume and a higher BBB permeability in the hippocampus of MCI.	[36]
**MCI**	WEPCAST	Increased BBB permeability to small molecules such as water but was not for large molecules such as albumin.	[3]
**Mild AD**	DCE-MRI	The BBB permeability was significantly higher in the total gray matter and cortex in AD patients compared with that in control subjects.	[32]
**Mild AD**	DCE-ASL-MRI	Reduced CBF and local blood volume in the gray matter of AD patients which was correlated with increased BBB leakage rate.	[33]
**AD**	Dual-time resolution DCE-MRI	BBB leakage was higher in the cortex but not white matter of AD patients	[35]
**AD**	^18^F-FDG PET	^ 18^F-FDG uptake reductions in the parietotemporal regions, posterior cingulate cortex, and hippocampus in AD patients.	[42]
**EOAD and LOAD**	^18^F-FDG PET	A significant decrease in glucose consumption in a wide portion of the left parietal lobe in EOAD compared with LOAD.	[43]
**FAD**	^18^F-FDG PET	Glucose metabolism reductions were found in the whole brain, bilaterally inferior parietal lobule, superior temporal gyrus, entorhinal cortex, posterior cingulate cortex, and hippocampus of FAD compared with controls.	[44]
**Mild AD**	^11^C-verapamil PET	BBB P-glycoprotein activity was significantly lower in the parietotemporal, frontal, and posterior cingulate cortices and hippocampus of mild AD	[49]

Note: MRI: Magnetic resonance imaging; DP-ASL: Diffusion-prepared, arterial spin labeling; DCE: Dynamic contrast-enhanced; WEPCAST:Water-extraction-with-phase-contrast-arterial-spin-tagging; ^18^F-FDG PET:^18^F-fluorodeoxyglucose; PET: Positron emission tomography;EOAD: Early onset AD; LOAD: Late onset AD; FAD: Familial AD.

### 3.1 Neuroimaging evidence

Owing to rapid advances in neuroimaging technology, the measurement of BBB dysfunction in subjects with mild cognitive impairment (MCI) and early AD has been achieved [25, 26]. We reviewed recent MRI and PET studies evaluating BBB permeability and function in AD patients (Table 1). Dynamic contrast-enhanced magnetic resonance imaging (DCE-MRI) is the most widely used technique for evaluating the breakdown of the BBB. A previous study using DCE-MRI and postprocessing analysis demonstrated an age-dependent BBB breakdown in the hippocampus [27]. It was also shown that BBB dysfunction in the hippocampus was correlated with pericyte injury [27]. Another study showed that the BBB permeability in the total gray matter and cortex of early AD patients was significantly increased compared with that in control subjects using the DCE-MRI sequence [28]. Another study used DCE-MRI and arterial spin labeling MRI to measure BBB permeability and CBF and showed that early AD patients demonstrated significant CBF reduction and BBB impairment [29]. More recently, a study demonstrated that the BBB had increased permeability to small molecules (i.e., water) but not to large molecules in MCI patients [3]. Another study showed that water exchange across the BBB is associated with a low CSF Aβ42 concentration in healthy older adults [30]. Similar results showing increased BBB permeability in AD patients were also found in several other previous studies [31-33]. However, specific and reliable measurement of low-level BBB permeability remains a great challenge and better methodologies need to be established [34].

BBB transporter abnormalities, which are another aspect of BBB dysfunction, have also been identified in MCI and early AD patients [35]. Glucose is the most important energy substrate for the brain. Glucose transporter expression in the endothelium of the BBB is required for brain uptake of glucose [36]. The most frequently used technique to evaluate glucose transport in the BBB is ^18^F-fluorodeoxyglucose positron emission tomography (^18^FDG-PET). Brain uptake of FDG depends on the expression of glucose transporters in the endothelium of the BBB [37]. Thus, the reduced uptake of FDG in AD patients reflects impaired BBB function. A previous multicenter study that performed ^18^FDG-PET in patients with AD, normal aging, and other neurodegenerative diseases, including frontotemporal dementia (FTD) and dementia with Lewy bodies (DLB), demonstrated that most of these patients displayed disease-specific PET patterns [38]. In addition, the decreased glucose uptake in the brain was also observed in early-onset AD [39] and presymptomatic familial AD patients [40]. Furthermore, reduced ^18^FDG-PET was reported as a risk factor for the conversion of MCI patients to AD dementia in several longitudinal studies, suggesting that BBB damage was associated with AD disease progression [41, 42]. More recently, a study examined ^18^FDG-PET/MRI in AD, MCI, and normal controls. The results demonstrated significant structural and metabolic alterations in the hippocampus of AD and MCI patients compared with normal healthy controls [43]. Another efflux transporter expressed in the endothelium of the BBB is P-glycoprotein, which is also abnormal in AD patients. The function of P-glycoprotein can be evaluated using ^11^C-verapamil-PET [44]. Previous studies have shown reduced P-glycoprotein activity in several brain regions of AD and MCI patients [45, 46]. Notably, P-glycoprotein is upregulated in the early stage of AD to assist Aβ clearance across the BBB. However, accompanied by disease progression, Aβ deposition and consequent events such as neuroinflammation, oxidative stress, and mitochondrial dysfunction, impaired P-glycoprotein expression, and function and exacerbated the deposition of Aβ [47].

In addition, several studies aimed to evaluate BBB dysfunction in transgenic AD models expressing mutations in human AD causative genes have also been conducted in recent years. One previous study found that increased BBB water permeability in the TgF344-AD transgenic rat model that expressed human amyloid precursor protein (APP) and presenilin 1(PSEN1) gene mutations using multiple flip angles multiple repetitions (MFAME) MRI [48]. Similarly, another study performed PET imaging found elevated neutrophil infiltration in the AD triple-mutant transgenic mic model, suggesting a higher blood-brain barrier permeability [49]. However, another study failed to identify signs of evident BBB dysfunction used in the transgenic ArcAβ mice model [50], suggesting that more sensitive imaging techniques might help to further illustrate the BBB alteration in future studies.

#### 3.2.2 Postmortem evidence

Evidence from the analyses of postmortem brain tissues have also demonstrated BBB dysfunction in AD patients. The most commonly used measurements included brain infiltration by circulating cells, leakage of blood-derived proteins such as fibrinogen, thrombin, and albumin, and structure changes in the BBB composition. Several studies used postmortem brain tissue from AD patients to identify brain infiltration by circulating cells. The results revealed the infiltration of peripheral macrophages in the brains of AD patients [51, 52]. It is believed that the increased activities of innate immune responses in the brain and the disrupted BBB facilitated the entrance of peripheral macrophages to the brain [53, 54]. In addition, analyses of postmortem brain tissues were used to determine the blood-derived proteins in the cortex that could reflect BBB breakdown. Depositions of fibrinogen, thrombin, albumin, and IgG were found in the hippocampus, prefrontal cortex, and entorhinal cortex of AD patients [55-57]. Furthermore, BBB structure alterations were also observed in AD patients. Previous studies have reported that increased BBB permeability could be caused by a loss or misalignment of TJ-related proteins or the loss of pericytes [58, 59]. Pericytes are important constituents of the BBB and play a critical role in its regulation [60, 61]. Decreased pericyte numbers in the hippocampus and cortex of AD patients were also observed [62], and these changes were most pronounced in patients carrying the *APOE* ε4 allele, suggesting that *APOE* participated in the BBB dysfunction [63, 64].

Pathology evidence from AD transgenic mouse models about cerebrovascular and BBB dysfunction was also frequently reported which had been well reviewed by a previous study [65]. Recently, one study reported that cerebrovascular changes featured with BBB permeability alteration, and PDGFRβ(+) pericytes decrease were observed in a 5XFAD mice model that expressed human *APP*, and *PSEN1* gene mutations [66]. Another study evaluated the effects of tau pathology on the BBB in P301L transgenic mice, and abnormal BBB morphology, decreased blood vessel diameters, and increased blood vessel density in the cortex were observed and were accompanied by cortical atrophy and altered expression levels of angiogenesis-related genes [67]. Similar results were found in an AD transgenic rat model with the APP_swe_ and PS1_Δe9_ mutations (TgF344-AD). Higher BBB water permeability was found in aged 13 months TgF344-AD rats and was related to AD pathology [68].

#### 3.2.3 CSF evidence

Cerebrospinal fluid biomarkers of BBB dysfunction have been identified in recent decades. The ratio of CSF albumin to serum albumin levels (the albumin quotient, Q-Alb) is frequently used to evaluate the integrity of the BBB [69]. The albumin quotient was elevated in AD and MCI patients in several previous studies [70], although a meta-analysis study demonstrated that Q-Alb was not a suitable biomarker for AD diagnosis [71]. CSF albumin levels can be affected by several factors such as CSF reabsorption and/or production, proteolytic cleavage, and the amount of CSF taken up by brain macrophages [23]. Thus, it is better to use other measurements together with Q-Alb when evaluating BBB permeability. Recently, several studies showed elevated CSF levels of soluble platelet-derived growth factor receptor-β (sPDGFRβ) in AD and MCI patients [72, 73], and this factor could be used as a biomarker for brain capillary damage and BBB breakdown [74]. However, these results require further replication.

## 4. The pathogenic mechanisms of BBB breakdown result in AD

### 4.1 BBB dysfunction and Aβ pathology

It is still controversial whether BBB dysfunction is a cause or consequence of Aβ pathology. As one of the core pathogenic hallmarks of AD, Aβ has attracted tremendous attention in recent decades. Aβ is believed to trigger tau hyperphosphorylation, microglial activation, oxidative stress, synaptic loss, and neuroinflammation. BBB breakdown was initially identified in AD genetic animal models with Aβ pathology. Aβ accumulates around the brain vasculature in 90% of AD cases, a condition called cerebral amyloid angiopathy [75]. Many studies have demonstrated that Aβ deposition increases BBB disruption [47]. The deposition of Aβ contributes to expression changes in TJ-related proteins, receptors such as advanced glycation end products (RAGE), and matrix metalloproteinases (MMPs), all of which play vital roles in BBB integrity [76-78]. Similarly, one study demonstrated the alteration of TJ-related proteins expression and efflux properties in brain endothelial cells, which derived from the induced pluripotent stem cells (iPSCs) of familial AD patients [79]. Additionally, Aβ induces oxidative stress and inflammation, resulting in pericyte degeneration and injury to BBB integrity [80].

BBB dysfunction also increases Aβ pathology. It was reported that BBB disruption exacerbated AD pathology by inhibiting P-glycoprotein and breast cancer resistance protein (BCRP) in a transgenic mouse model of AD [81]. Excess deposition of Aβ in AD is caused by an imbalance between its production and clearance. As a key structure involved in the clearance of waste metabolites, BBB dysfunction affects the Aβ clearance [82]. RAGE in the BBB promotes Aβ influx, whereas lipoprotein receptor-related protein (LRP) and P-glycoprotein facilitate the efflux of Aβ out of the brain [83]. The abnormality of these transporters in the BBB will cause Aβ accumulation in the brain and will facilitate AD pathology [84]. In addition, abnormal alterations of other transporters will cause abnormal metabolism of glucose and essential amino acids in the brain, which may aggravate the deposition of Aβ and contribute to the progression of AD. Previous studies have also shown that disruption of the BBB causes Aβ deposition by inducing inflammation [47]. Notably, Aβ and tau pathology were reduced when BBB integrity was restored in an AD transgenic mouse model by treatment with Annexin A1, indicating that the BBB was a potential therapeutic target for AD treatment [85].

### 4.2 BBB dysfunction and tau pathology

Another major histological hallmark of AD is neurofibrillary tangles (NFTs), which consist of aggregated abnormal P-tau. BBB breakdown is also observed in tauopathies without Aβ pathology, suggesting that tau is sufficient to induce BBB damage [86]. The effects of tau pathology on neurovascular function in AD are less studied than those of Aβ. Recently, a growing body of literature has shown that BBB integrity and functionality are affected by pathological tau, which subsequently promotes progression of the disease [87]. The tau and P-tau concentrations in CSF and plasma are significantly higher in AD patients than those in controls, and the toxicity of P-tau may be the chief contributor to neurovascular damage. BBB dysfunction is associated with the perivascular level of tau around hippocampal blood vessels [86]. More importantly, the progression of BBB damage was suppressed when tau expression was reduced, suggesting that the stability of the BBB can be modulated by tau levels [86]. Previous studies have reported that disruption of the BBB caused by tau pathology is driven by several mechanisms including mitochondrial dysregulation, oxidative stress, and chronic neuroinflammation. Tau pathology impacts mitochondrial localization, distribution, and dynamics, which further result in alterations in ATP and reactive oxygen species production [88]. In addition, tau pathology can directly modify the BBB and affect the immune cells that across the BBB [89]. Tauopathies also mediate the production of cytokines, chemokines, and adhesion molecules secreted by glial cells, neurons, and endothelial cells [90]. Mitochondrial dysregulation and inflammatory processes facilitate structural changes in capillaries and increase BBB permeability and BBB transporter dysfunction [91, 92].

The BBB prevents the free passage of tau into the blood. Transport of tau and P-tau across the BBB requires specialized transporters. Although specific BBB transporters for P-tau have not been identified, it is believed that BBB dysfunction influences the clearance of P-tau and consequently causes the accumulation of tau pathology [93].

### 4.3 BBB dysfunction and neuroinflammation

The role of BBB dysfunction and neuroinflammation in the pathogenesis of AD is complicated. After being activated by damage associated molecular patterns (DAMPs) such as Aβ through pattern recognition receptors (PRRs) or ATP, microglia and astrocytes produce proinflammatory cytokines such as interleukin 1 beta (IL-1β), IL-18, and Tumor necrosis factor α (TNF-α) [94]. Proinflammatory cytokines result in endothelial cells upregulating the expression of MMP-9, which is believed to degrade TJs and the extracellular matrix [95-97]. Following cytokine stimulation, the expression of TJ proteins decreases, leading to BBB breakdown. IL-1β depresses the expression of occludin, ZO-1, and claudin-5 through the p38, mitogen-activated protein kinase (MAPK), protein kinase C (PKC), and β-catenin/forkhead box O1 (FoxO1) pathways [98]. In addition to proinflammatory cytokines, the complement system also plays a critical role in BBB dysfunction. The activation of C3a receptors in brain endothelium cells causes increased BBB permeability that was dependent on intracellular Ca2+ levels in an aging model. Increased intracellular Ca2+ disrupts vascular endothelial cadherin-based adherens junctions, resulting in increased BBB permeability [99].

BBB breakdown, in turn, enhances the inflammatory response in the brain. Under physiological conditions, the brain is considered an immune-privileged place that restricts the infiltration of peripheral immune cells and plasma proteins due to a functional BBB. However, under pathological conditions including AD, stroke, traumatic injury, and other neurodegenerative diseases, increased BBB permeability allows immune cells to enter the brain parenchyma. Higher neutrophil infiltration was identified in the hippocampus of transgenic AD mice compared with that in wild-type mice, and these cells produced neurotoxic cytokine IL-17 and neutrophil extracellular traps (NETs) [100]. CD8+ T cells were observed in the brain parenchyma of APP/PS1 mice and affected synapse-related gene expression [101]. Inconsistent with animal experiments, some postmortem tissue studies have confirmed the existence of peripheral leukocytes and macrophages in the brain parenchyma or perivascular space in patients with AD [22]. More recently, scientists discovered CD8+ T cells in the CSF of AD patients via mass cytometry and single-cell analysis [51]. Using a similar strategy, T cells were discovered in aged brains and inhibited the proliferation of neural stem cells [102].

Two families of adhesion molecules mediate the infiltration of peripheral immune cells: the integrins family and the selectins family. Very late antigen-4 (VLA-4) and lymphocyte function-associated antigen 1 (LFA-1) belong to the integrin family. Their ligands are vascular cell adhesion molecule-1 (VCAM-1) and intracellular cell adhesion molecule-1 (ICAM-1) [103]. The second family is composed of L-selectin, E-selectin, and P-selectin [104]. Higher levels of soluble adhesion molecules were found in the plasma of AD patients and were correlated with more severe dementia [105]. Animal experiments confirmed that the adhesion molecules VCAM-1 and LFA-1 played an important role in cognitive impairment [100, 106]. Using two-photon laser scanning microscopy (TPLSM) experiments, one study demonstrated that neutrophils were present adjacent to Aβ deposits, and Aβ triggered LFA-1 into a high-affinity state which enhanced neutrophil infiltration [100].

In addition to immune cells, plasma proteins that infiltrate the dysfunctional BBB such as fibrinogen/fibrin can induce neuroinflammation. Fibrin binds to CD11b/CD18 in microglia or infiltrated macrophages, activating the MAPK, NF-κB, and phosphoinositide 3-kinase (PI3K) pathways which mediate adhesion, migration, chemotaxis, and phagocytosis. In addition, fibrin binding to CD11b/CD18 also triggers the conversion of resting microglia to M1 microglia and produces neurotoxic cytokines and reactive oxygen species (ROS) [107]. In recent years, pericytes have attracted tremendous attention for their roles modulating neuroinflammation [108]. Their roles in BBB formation and CBF regulation have been well described, although how they contribute to neuroinflammation remains controversial [109]. During inflammatory stimulation, pericytes produce large quantities of immune mediators such as proinflammatory cytokines (L-1β, IL-18, and TNF-α), ROS, chemokines (CCL2), adhesion molecules (ICAM-1, and VCAM-1), and MMPs (MMP-2, and MMP-9) [109]. As a result, the immune mediators induced by pericyte activation cause TJ degradation, neuronal death, leukocyte recruitment, and infiltration. Notably, pericytes are also involved in the anti-inflammatory process. Several anti-inflammatory components are produced by pericytes including IL-33 [110]. IL-33 was confirmed to ameliorate AD pathology and cognitive decline in APP/PS1 mice [111]. A recent study also demonstrated that pericytes restrict the infiltration of leukocytes [15]. The diverse roles of pericytes in neuroinflammation might result from different cell types or different conditions. The specific mechanisms need to be further elucidated in the future.

## 5. The implications of BBB dysfunction in AD for delivering therapeutic drugs

The systemic delivery of molecular cargo into the brain is regulated by the BBB. Four basic mechanisms for drug delivery have been proposed, including passive diffusion, active efflux transport, carrier-mediated transport, and receptor-mediated transport. Passive transport refers to the spread of molecules through the biofilm along their concentration gradient, without the expenditure of biological energy or involvement of a carrier protein [112]. More specifically, this transport can be divided into paracellular (i.e., TJ), transcellular (i.e., lipophilic molecules), or aqueous channels (i.e., hydrophilic molecules) [113]. Physical properties such as the size and hydrophobicity of molecules are the main determinants of different passive diffusion paths. The active efflux system in the BBB is mainly composed of P-glycoprotein and BCRP [114]. Both belong to the ATP-binding cassette (ABC) superfamily of transporters and limit therapeutic drug entry into the brain. Carrier-mediated transport (CMT) is involved in the transport of low molecular weight (MW) organic molecules or essential nutrients (i.e., the transport of glucose through GLUT) [115]. The receptor-mediated transport (RMT) system includes insulin receptors, transferrin receptors, low-density lipoprotein (LDL) receptors, and several others all of which are important for macromolecular endocytosis regulation [116].

Under physiological conditions, TJs, and active efflux transport inhibit the entry of almost 98% of small molecules and 100% of macromolecules [117]. In the context of AD, the effect of BBB evolution on drug delivery is more complicated. On the one hand, the loss of TJs, increase permeability of the BBB, which may promote drug delivery to the brain [22, 118]. However, leaky blood-borne molecules and cells may interfere with drug distribution throughout the brain [22, 119]. Simultaneously, decreased numbers of receptors or carriers in a diseased BBB (i.e., LRP1 and GLUT1) may obstruct molecular transport between the center and the periphery. Therefore, based on the altered BBB in AD, there are a series of strategies for drug delivery. We summarize the following strategies against BBB dysfunction.

### 5.1 TJ loss-based strategies

TJ loss in the diseased BBB may improve drug delivery. However, a previous study found that CNS penetration of small molecule drugs was not promoted in the AD animal model [120]. It is estimated that even under severe pathological conditions, only molecules smaller than 20 nm can penetrate the BBB through this pathway[121, 122]. Fortunately, nanotechnology may overcome this huge obstacle. Liposomal and polymeric nanoparticles are two classic agents, and both are reported to be potential strategies for AD therapy [123]. Carbon dots, which are novel nanoparticles that are only in 1-10 nm size, have been verified to have good BBB penetration and Aβ fibrillation inhibition [124]. In addition to relying on a disrupted BBB, BBB modulators (i.e., lexiscan and minoxidil) can be loaded into nanoparticles to improve drug delivery [125-127].

### 5.2 CMT and RMT based strategies

Based on the characteristics of the BBB under pathological conditions, the design of a modified brain drug delivery system is of great significance for treatment. In AD, RAGE upregulation occurs in the early stage [128], and RAGE mediated transcytosis can be utilized for drug delivery. Gospodarska et al. found the binding domain of Aβ and RAGE [129], and Lu et al. designed a small peptide-modified polymeric micelle system based on the Aβ sequence [130]. This system improved the efficiency of CNS drug delivery in the AD mouse model. Other common receptors that mediate molecular transport including transferrin receptor [131-135], LDL receptors [136, 137], and insulin receptor [138] have also been described as targets of nanomedicine for AD treatment. However, several carriers or receptors are downregulated in AD (i.e., LRP1 and GLUT1), which exacerbates Aβ aggregation and hinders drug delivery. Statins and glucose can be used to upregulated LRP1 and GLUT1 at the BBB [139, 140], respectively. Although it has not been applied to AD treatment, this strategy has great potential for brain-targeted drug delivery.

### 5.3 Blood-borne leaky based strategies

The leakage of peripherally derived cells into the brain through the disrupted BBB provides a new opportunity for cell membrane-coated nanotechnology in AD treatment. This biomimetic strategy shows great biocompatibility, prolonged circulation, and CNS-targeting. Currently, the most frequently used cell membrane in AD therapy is the red blood cell (RBC) membrane [141-144]. Han et al. designed an RBC membrane-coated nanoparticle modified with RVG29 and triphenylphosphine cation (TPP) molecules to achieve BBB penetration and mitochondrial targeting [144]. This system rescued ROS-induced mitochondrial dysfunction and improved memory impairment in APP/PS1 mice. In the future, other biofilms such as macrophage membranes, platelet membranes, modified cell membranes [145], and exosome membrane [146, 147] may be considered candidates for this strategy.

## 6. Conclusions and future directions

**Figure 2.**
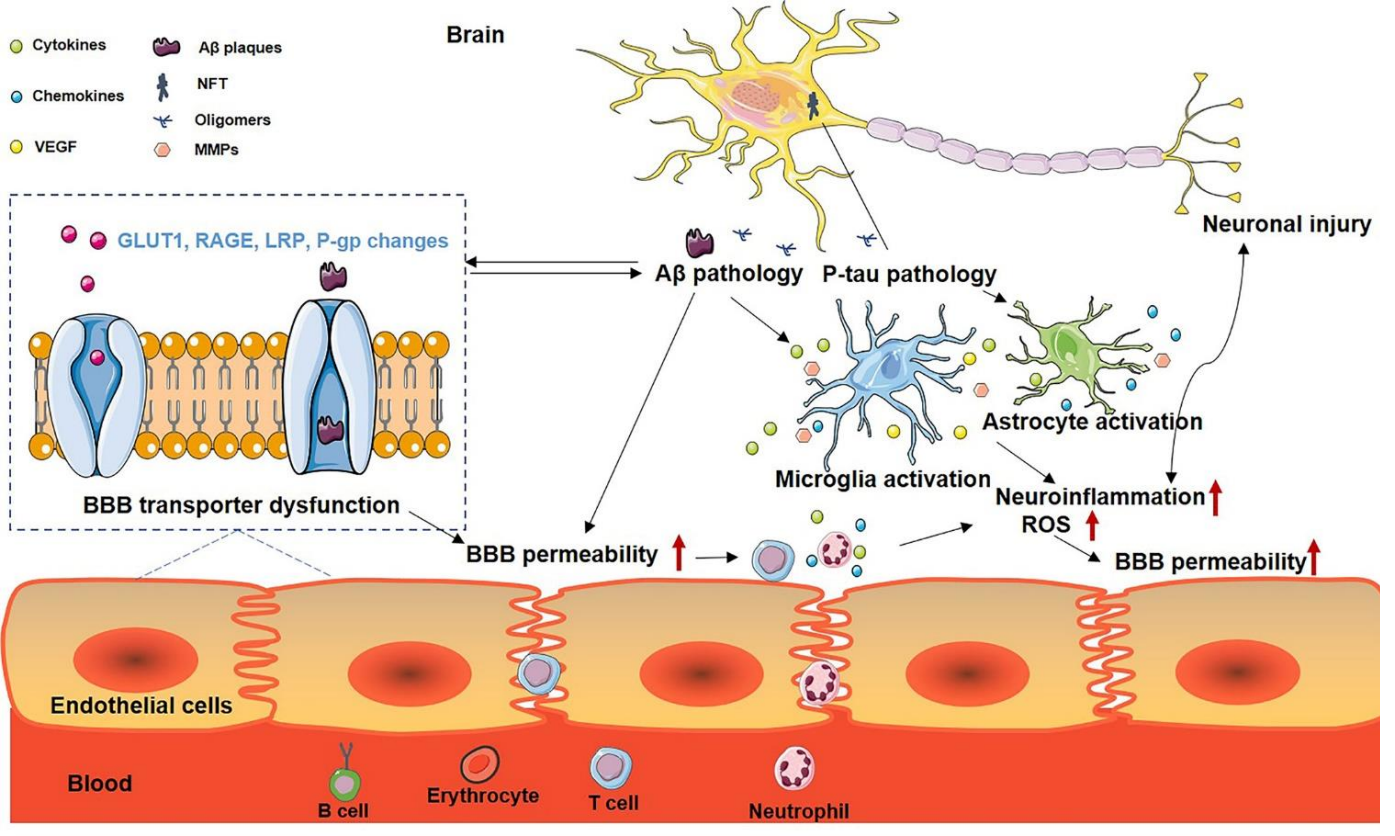
**The proposed model of blood-brain barrier (BBB) disruption in Alzheimer’s disease focuses on its interaction between amyloid-β (Aβ), hyperphosphorylated tau (P-tau), and neuroinflammation**. The Aβ and the P-tau pathology lead to microglia and astrocyte activation. Activation of microglia and astrocyte and elevated reactive oxygen species (ROS) level would facilitate the processes of neuroinflammation and consequently induce BBB breakdown, including increased BBB permeability, BBB transporters dysfunction, and abnormality of proteins secretion by BBB cellular components. On the other hand, BBB permeability alteration facilitates the migration of inflammatory cells and molecules into the brain, subsequently promoting AD pathology. The dysfunction of the transporters in BBB lead to the impairment of Aβ, tau clearance, results in the deposition of Aβ and formation of NFT.

In conclusion, we have summarized the evidence for the roles of BBB disruption in AD pathogenesis and the pathogenic mechanisms by which BBB dysfunction results in AD onset and neurodegenerative processes. We have discussed the reciprocal relationships between BBB dysfunction, Aβ pathology, tau pathology, and neuroinflammation (Fig. 2).

Although the disrupted BBB has been reported to contribute to the initiation and progression of AD, further study is needed to illustrate the precise causative factors (such as genetics, environment, and vascular risk factors) and the molecular mechanisms of BBB dysfunction underlying the pathogenesis of AD. High-quality clinical studies that determine the associations between BBB breakdown and AD as well as the conditions of normal aging will help us better understand the value of BBB integrity in early AD diagnosis and disease progression monitoring. In this regard, the development of brain imaging techniques that could specifically, reliably and non-invasively measure low-level BBB permeability or transporters changes are particularly important. Damage to the BBB integrity and the consequent infiltration of serum components into the brain can facilitate a multitude of processes resulting in progressive synaptic, injury, neuronal injury, and neuroinflammatory changes. BBB repair may reduce the pathology of Aβ, P-tau, and neuroinflammation. Restoration of BBB disruption may represent a promising target for the treatment of AD. Further efforts are needed to the reconstruct the BBB and determine its role in AD prevention and treatment. Potential approaches include the rescue of the TJs of endothelial cells, restoring efflux transporters, and rescuing pericyte degeneration.
